# Clinical Relevance of Nontuberculous Mycobacteria Isolated from Sputum in a Gold Mining Workforce in South Africa: An Observational, Clinical Study

**DOI:** 10.1155/2015/959107

**Published:** 2015-05-28

**Authors:** Clare L. van Halsema, Violet N. Chihota, Nicolaas C. Gey van Pittius, Katherine L. Fielding, James J. Lewis, Paul D. van Helden, Gavin J. Churchyard, Alison D. Grant

**Affiliations:** ^1^London School of Hygiene and Tropical Medicine, Keppel Street, London WC1E 7HT, UK; ^2^The Aurum Institute, Postnet Suite No. 300, Private Bag X30500, Houghton, Johannesburg 2041, South Africa; ^3^DST/NRF Centre of Excellence for Biomedical Tuberculosis Research, MRC Centre for Molecular and Cellular Biology, Division of Molecular Biology and Human Genetics, Faculty of Medicine and Health Sciences, Stellenbosch University, Private Bag X1, Matieland, Cape Town 7602, South Africa; ^4^School of Public Health, University of Witwatersrand, Private Bag 3, Wits 2050, South Africa

## Abstract

*Background*. The clinical relevance of nontuberculous mycobacteria (NTM), detected by liquid more than solid culture in sputum specimens from a South African mining workforce, is uncertain. We aimed to describe the current spectrum and relevance of NTM in this population. *Methods*. An observational study including individuals with sputum NTM isolates, recruited at workforce tuberculosis screening and routine clinics. Symptom questionnaires were administered at the time of sputum collection and clinical records and chest radiographs reviewed retrospectively. *Results*. Of 232 individuals included (228 (98%) male, median age 44 years), *M. gordonae* (60 individuals), *M. kansasii* (50), and *M. avium* complex (MAC: 38) were the commonest species. Of 38 MAC isolates, only 2 (5.3%) were from smear-positive sputum specimens and 30/38 grew in liquid but not solid culture. MAC was especially prevalent among symptomatic, HIV-positive individuals. HIV prevalence was high: 57/74 (77%) among those tested. No differences were found in probability of death or medical separation by NTM species. *Conclusions*. *M. gordonae, M. kansasii*, and MAC were the commonest NTM among miners with suspected tuberculosis, with most MAC from smear-negative specimens in liquid culture only. HIV testing and identification of key pathogenic NTM in this setting are essential to ensure optimal treatment.

## 1. Introduction

The nontuberculous mycobacteria (NTM) form a group of organisms diverse in many characteristics, including pathogenicity and clinical disease. They have environmental reservoirs and are associated with a broad spectrum of clinical presentations from cutaneous to lung or disseminated disease. There is uncertainty regarding clinical relevance of many of the species, particularly in settings where facility to isolate and identify them is relatively recent.

Liquid mycobacterial culture is recommended for investigation of suspected tuberculosis in resource-limited settings [1]. Compared with solid culture, time to positive culture is decreased and yield increased [2–4]. NTM accounted for 77% of the additional yield from liquid versus solid culture of smear-negative sputa in a gold mining population [2]. It is therefore important to understand the clinical relevance of NTM isolates, particularly in settings of high HIV and tuberculosis prevalence, where NTM may be isolated from large numbers of individuals in tuberculosis programmes. To date, there is limited information from such settings, as culture and speciation facilities have not been widely available or generally applied [5].

Gold miners in South Africa have a very high incidence of tuberculosis [6] and high prevalence of HIV (estimated at 29% in 2000-2001 [7]) and silicosis [8, 9]. Previous studies in this population demonstrated disease due to NTM, particularly* M. kansasii*,* M. scrofulaceum*, and* M. avium* complex [8, 10–13]. We have previously reported on NTM species identification in this population [14] and now describe the clinical features of those with NTM isolated from sputum, to inform laboratory and clinical practice. Previous work on molecular characteristics of* M. gordonae* isolates [14] led to the specific objective of reevaluating the clinical relevance of* M. gordonae* in this setting. We used a cross-sectional, observational study design, in the context of “Thibela TB,” a cluster randomised trial of community-wide isoniazid preventive therapy (IPT) [15, 16].

## 2. Methods

**Figure 1:**
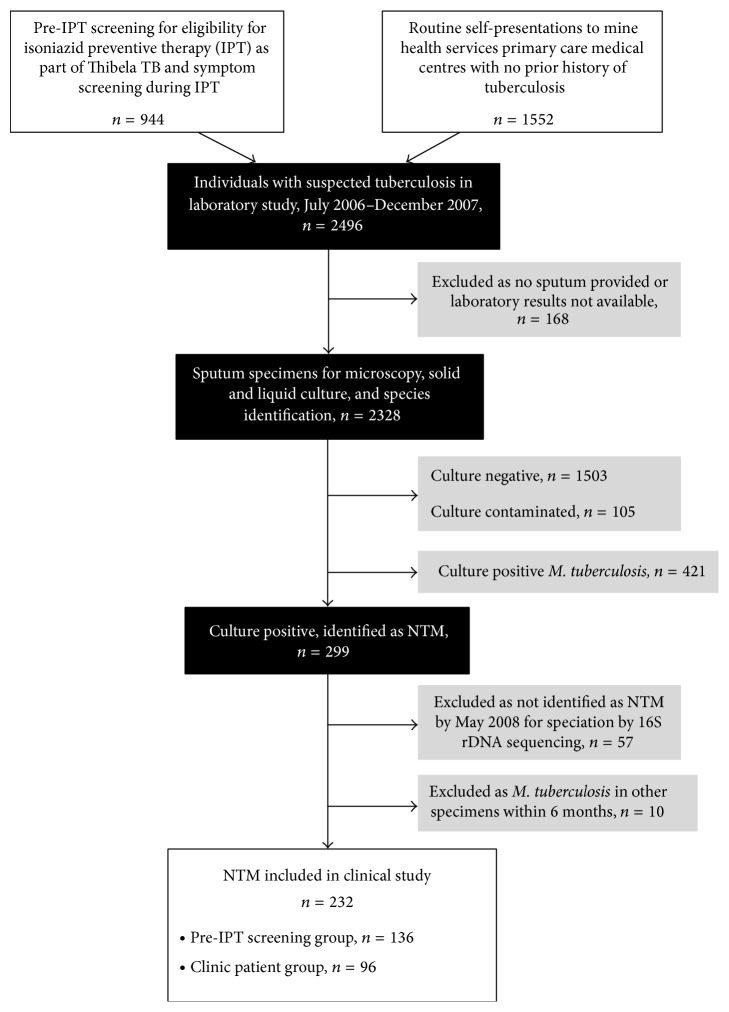
Flow chart showing sources of study participants.

For a laboratory study evaluating mycobacterial culture media [2], participants were recruited from two sources (Figure 1).

1. Screening for active tuberculosis at enrolment to Thibela TB (pre-IPT screening group): enrolment was open to the whole workforce. Screening was by symptom questionnaire and chest radiograph; nine months' IPT was given to those with negative screen. If tuberculosis was suspected at screening, sputum was taken for mycobacterial culture. All those providing sputum, regardless of tuberculosis history, were eligible for this laboratory study.
2. Clinics run by mine health services (clinic patient group): individuals self-presented with symptoms or were referred from occupational health services, which conducted radiological screening of all employees every 6–12 months. Recruitment was restricted to individuals with no prior history of tuberculosis, aiming to strengthen diagnostic services for this group who would not routinely have been investigated with sputum culture.

Participants, recruited from July 2006 to January 2009, gave one sputum specimen, after nebulisation if necessary, and were interviewed using a standardised symptom questionnaire, which was more detailed for the clinic patient group. For this study, we included all those with NTM from sputum collected up to the end of December 2007 and with species identification using standard biochemical testing (SBT) completed by May 2008. Those with* M. tuberculosis* isolated within six months of the index NTM were excluded as clinical disease was likely to be due to tuberculosis. The pre-IPT screening and clinic patient groups are described separately, as characteristics of the two groups were expected to differ in terms of symptoms and stage of disease. Study participants with suspected tuberculosis were referred to the mine health service for subsequent investigation and treatment; no additional management of patients was done by investigators and this study was entirely observational, reporting routine practice in this setting.

### 2.1. Laboratory Methods

As described previously [2, 14], sputum smears were examined for acid fast bacilli by fluorescence microscopy; 0.5 mL sediment was cultured in BACTEC MGIT 960 system (BD Diagnostic Systems, Sparks, MD, USA) and a further 0.5 mL on Löwenstein-Jensen slants, which were incubated for up to eight weeks at 37° Celsius. Mycobacteria were identified by SBT as previously described [14], anti-MPB64 assay (TAUNS, Numazu, Japan) and microscopic cording. Subsequently, NTM underwent DNA sequence amplification (using 45 amplification cycles) and analysis of the hypervariable regions of the 16S rRNA gene by PCR, with referral to the RIDOM and GenBank databases for identification [17, 18]. For specimens in which chromatograms indicated mixed NTM species, the main species is reported; numbers with mixed species are given in Table 1. A new species was defined as an isolate whose sequence did not match a known sequence in the reference databases.

### 2.2. Clinical Record Review

Medical and laboratory records, accessed at primary health care centres and mining hospitals, were reviewed retrospectively, using a standardised case report form in order to report routine clinical practice in this setting. Mine health services policy is to offer HIV tests to those diagnosed with tuberculosis: tests were offered to individuals in this study according to medical staff practice, with no testing done for research purposes.

### 2.3. Chest Radiographs

The chest radiograph closest to sputum specimen collection was read using a standardised reporting form, recording features consistent with tuberculosis, along with a silicosis score according to the International Labour Office guidelines [20]. Signs were classified as consistent with definite, probable, possible, or no disease for both active and previous tuberculosis, according to the judgment of the reader, following guidance of the chest radiograph reading and recording system [21]. The reader was masked to clinical details. For the pre-IPT group, an additional 10% of radiographs were added to further mask the reader, from those who did not have NTM isolated (no isolates or* M. tuberculosis*).

### 2.4. Follow-Up

Individual records were linked to mine company human resources records to establish employment and vital status of participants. Survival analysis was used to examine proportions remaining in the workforce by NTM species isolated, for majority species, from the date of sputum specimen collection. Participants were followed up from the date of sputum specimen collection until the earliest of death, medical separation (individuals leaving the workforce for medical reasons), leaving employment, or censoring date (31 December 2010).

### 2.5. Definitions

Individuals were considered to be HIV-positive if a positive HIV test was recorded before or up to one year following enrolment and HIV-negative if a negative HIV test (and no subsequent positive test) was recorded after or up to one year preceding enrolment. Treatment outcomes for those treated with standard tuberculosis regimens were recorded according to WHO definitions [22]. Members of the* M. avium* complex were grouped together for the most of the descriptive work presented here.

### 2.6. Statistical Methods

Categorical variables were compared using chi-squared or Fisher's exact test and continuous variables using the Kruskal-Wallis test. The log rank test and Kaplan-Meier curves were used to compare proportions remaining in the workforce by NTM species.

### 2.7. Ethical Approval

This study was approved by the Research Ethics Committees of the University of KwaZulu-Natal and the London School of Hygiene and Tropical Medicine. All participants gave written or witnessed verbal consent.

## 3. Results

### 3.1. Study Population

From July 2006 to December 2007, 2496 individuals were recruited and provided sputa. 720 specimens (28.8%) yielded mycobacterial growth, as described previously [2]. Of these, 421/720 (58.5%) grew only* M. tuberculosis* and 299/720 (41.5%) grew NTM. Fifty-seven were excluded because species identification by SBT was not completed by May 2008; 10 were excluded because of concurrent* M. tuberculosis*. Of 232 individuals included (Figure 1), 136 were enrolled through pre-IPT screening and 96 were clinic patients.

Two hundred and twenty-eight (98.3%) of study participants were male, with median age of 44 years (interquartile range (IQR) 36, 49 years; median age 44 years [IQR 36–48] for pre-IPT group and 45 years [IQR 36–50] for clinic patient group), compared with 43 years (IQR 37, 49) in the parent laboratory study [2] and 40 (IQR 31, 46) among 23299 individuals enrolling into Thibela TB at intervention clusters [23]. Median time worked in mining was 21 years (IQR 10, 28). In the pre-IPT screening group, 63/136 (46.3%) had a history of prior tuberculosis treatment, compared with 10.7% of the 23299 enrolling at intervention clusters [23].

### 3.2. Mycobacterial Species and Sputum Smear Status

Species isolated are shown in Table 1, by recruitment route. In the clinic patient group, 25 individuals, two of whom were sputum smear positive, had* M. avium* complex (12* M. colombiense*; six* M. vulneris*; five* M. intracellulare*;one each of* M. avium *and* M. chimaera*).* M. kansasii* was isolated from 21 individuals, six of whom were sputum smear positive, and* M. fortuitum* from nine. Seven had new mycobacterial species. In the pre-IPT screening group,* M. gordonae* (53 individuals) was the commonest species, followed by* M. kansasii* (29 individuals) and then members of the* M. avium* complex (13 participants), principally* M. colombiense* (seven of 13). Eight of 53* M. gordonae*, 11 of 29* M. kansasii*, and none of the* M. avium* complex were isolated from smear-positive sputum specimens.

### 3.3. HIV Prevalence

Combining groups, HIV status was documented for 74/232 (31.9%) individuals (Table 1). HIV prevalence among those with* M. avium* complex was 12/15 (80.0%), for* M. kansasii* 14/22 (63.6%), and for* M. gordonae* 11/13 (84.6%). Median CD4 count among HIV-positive individuals with* M. avium complex* was 135 cells/*μ*L (range 14, 827; *n* = 9),* M. kansasii* 169 cells/*μ*L (range 39, 763; *n* = 13), and* M. gordonae* 291 cells/*μ*L (range 66, 396; *n* = 9). Species distribution by HIV status is shown in Table 2.

Of 57 HIV-positive individuals, 43 (75.4%) were attending HIV care and 8/43 (18.6%) had evidence of taking antiretroviral therapy.

### 3.4. Clinical and Radiological Features

Symptoms reported at the time of sputum specimen collection are shown in Table 3. In the clinic patient group, cough (17/25; 68.0%) and night sweats (13/25; 52.0%) were prevalent among those with* M. avium* complex. Cough was common for all NTM species and fever was reported most frequently by those with* M. parascrofulaceum* (6/9) and* M. gordonae* (4/7).

In the pre-IPT screening group, cough was less common than in the clinic patient group reported by 59/136 (43.4%) versus 66/96 (68.8%) individuals. Cough was reported by 8/13 (61.5%) of those with* M. avium* complex and 26/53 (49.1%) of those with* M. gordonae*.

Combining pre-IPT screening and clinic patient groups, of 60 individuals with* M. gordonae*, 21 (35.0%) reported weight loss and 14 (23.3%) night sweats, while, of 38 with* M. avium* complex, 18 (47.4%) reported night sweats and 14 (36.8%) weight loss.

Chest radiographs were available for 171/232 (73.7%) individuals. Prevalence of silicosis, grade 1/0 or above, was 38/171 (22.2%) and grade 1/1 or above 13/171 (7.6%).* M. kansasii* was most frequently associated with chest radiograph appearance suggestive of definite or probable active tuberculosis (17/34, 50.0%); corresponding figures for* M. avium* complex were 3/26 (11.5%) and for* M. gordonae* 14/49 (28.6%). Lung cavitation was present in 47/171 (27.5%) radiographs including 22/34 (64.7%) of those with* M. kansasii*, 11/49 (22.5%) with* M. gordonae*, 2/15 (13.3%) with* M. fortuitum*, and 2/26 (7.7%) with* M. avium* complex (chi^2^ *P* < 0.001 for comparison of four species). Among those with cavitation, this was considered to be due to active disease in 6/11 (54.5%) with* M. gordonae* and 8/22 (36.4%) with* M. kansasii*.

*M. gordonae* isolates with discrepant identification on SBT were further identified by strain, as described previously, with no differences seen between clinical features of those few isolates identified as strain D versus other* M. gordonae* strains [14].

### 3.5. Species Isolated by Culture Method

Of 38* M. avium* complex isolates, 30 (78.9%) were cultured on liquid but not solid culture medium. Of those 30, 9/12 individuals with known status were HIV-positive and none were sputum smear positive. For other species, 6/20 (30.0%)* M. fortuitum*, 38/60 (63.3%)* M. gordonae*, 12/50 (63.3%)* M. kansasii*, and 15/21 (71.4%)* M. parascrofulaceum* were isolated only on liquid medium (chi^2^ test, *P* < 0.001). Combining all species, no significant differences in reported symptoms were observed between those cultured on solid medium and those cultured only on liquid medium (data not shown).

### 3.6. Outcomes and Loss to Workforce

**Figure 2:**
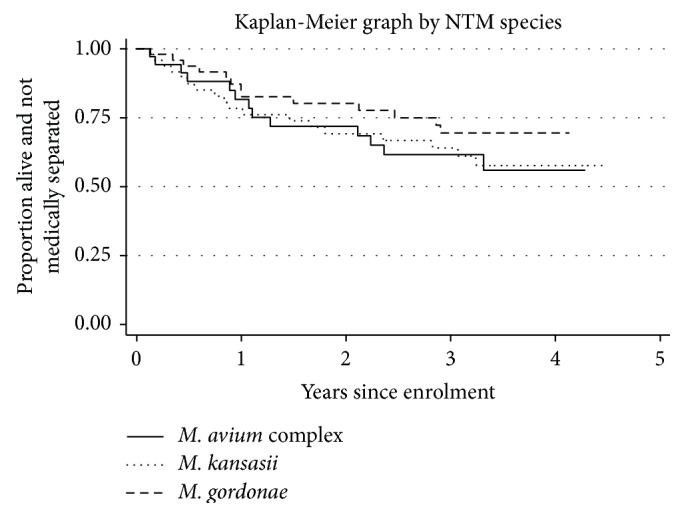
Kaplan-Meier graph showing death or medical separation by NTM species isolated for the three most prevalent species (*n* = 138: 51* M. gordonae*; 49* M. kansasii*; and 38* M. avium* complex).

Linking to human resources data was successful for 218/232 (94.0%) individuals. Median follow-up time was 31.9 months (range 0.07 to 54.7 months), during which time there were 10 deaths and 63 medical separations, as shown in Table 4. At 24 months, 80.3% with* M. gordonae*, 69.2% with* M. kansasii*, and 72.9% with* M. avium* complex remained in the workforce (Table 5). Comparing the three commonest species (*M. gordonae*;* M. kansasii; M. avium* complex), log rank test for differences in proportions remaining in the workforce gave *P* = 0.47 (Figure 2).

## 4. Discussion

In this mining population with high HIV prevalence, NTM were common in sputum culture of those with suspected tuberculosis.* M. gordonae, M. kansasii*, and* M. avium* complex were the commonest species, with* M. avium* complex particularly prevalent among symptomatic individuals presenting to routine health services and* M. gordonae* most common in the pre-IPT screening group.

HIV prevalence was 77.0% where known. Importantly, though this study population comprised individuals with suspected tuberculosis, a minority had HIV status documented, suggesting that testing coverage is poor and that those tested may not be representative of all those with NTM. It is possible that those tested have higher prevalence of HIV than those not tested, as there may have been clinical reasons for offering tests. However, routine testing in this setting is essential to improve care and it is very likely that diagnoses of HIV are being missed. Silicosis grade 1/1 or above (definite silicosis) was found in 7.6% of individuals, compared with 2.6% in the parent study Thibela TB [16], which would be expected in a subpopulation with NTM, as silicosis is a known risk factor [8].

Symptoms varied by recruitment method, as expected, with cough more prevalent among those self-presenting to routine health services than those undergoing screening. Lung cavitation was more prevalent among those with* M. kansasii* than those with* M. avium* complex, which would be expected, as* M. avium* complex more typically causes disseminated disease in the context of advanced HIV-related immunosuppression and sputum isolates may represent disseminated, rather than exclusively pulmonary, disease. It is not possible to definitively diagnose disseminated disease without further sampling, but, with low CD4 counts, systemic symptoms, and chest radiographs without cavitation, it is likely that some of this group had disseminated* M. avium* complex, with the organism isolated from respiratory specimens here. Similarly, radiographs were reported as consistent with active tuberculosis less frequently in those with* M. avium* complex. There was a higher than expected proportion with cavitation on chest radiograph among those with* M. gordonae*. This is difficult to interpret with limited clinical data available and cavitation may have been a result of previous or other diseases. We did not detect differences in proportions remaining in the workforce between groups of individuals with the three commonest NTM species, although there is a suggestion of lower retention in the workforce among those with* M. avium* complex and* M. kansasii*. The study design did not include a comparator group without NTM and from these data alone we can only conclude that loss to the workforce for health reasons among those with NTM isolated from sputum is substantial. However, from the mining population participating in Thibela TB, mortality was 0.91/100 person-years and combined mortality and medical separation were 4.34/100 person-years [16], suggesting higher combined mortality and medical separation among those with NTM than the workforce overall, which may be due to mycobacterial disease or untreated HIV. Differences by species may have been detected with larger numbers.

The distribution of NTM species differs from that found in previous studies in this population. In the 1990s,* M. kansasii* and* M. scrofulaceum* were reported to be the commonest species [8, 11], whereas this study found higher proportions of* M. avium* complex and* M. gordonae*, with notable absence of* M. scrofulaceum* using 16S sequencing. This is likely to be largely due to newer speciation methods: organisms identified by SBT as* M. scrofulaceum* in this study were almost all subsequently identified as* M. gordonae* by 16S sequencing [14]. The higher proportion of* M. avium* complex may be due to the use of liquid culture, as suggested by laboratory studies in this population [2] and elsewhere [3, 4], and may also be due to increased HIV prevalence in this population: from 1.4% among those with sexually transmitted infections in 1991 [24] to 19.0% among those with NTM in 1993-6 [11, 25] and 29% in a representative sample of the workforce in 2000-1 [7]. The median age of the population does not appear to have increased, estimated at 41 years in 2001 [7] and 40 years among those enrolling at Thibela TB intervention clusters [23], although the median age among those with NTM was higher.

*M. gordonae* was more prevalent in the pre-IPT screening group than in clinic patients, perhaps because, in the context of active case finding, not all isolates will be genuine pathogens.* M. gordonae* is well documented to be a laboratory and tap-water contaminant [19]. However, in this group of individuals with suspected tuberculosis,* M. gordonae* was associated with systemic symptoms (night sweats, weight loss) in some, with radiological abnormalities in 14/51 (27.5%), and with HIV infection in 11/15 (73.3%). It has previously been reported to cause disseminated disease in advanced HIV [26–28] and may be pathogenic in some individuals here, with isolates from sputum indicating pulmonary or more widespread disease. Laboratory and clinical records would be expected to have detected concurrent* M. tuberculosis* or bacterial infections if present. Longer term, close follow-up of these individuals was not incorporated into the study design. Without more clinical data and follow-up, firm conclusions on* M. gordonae* in this population should not be drawn, but those with* M. gordonae* and clinical symptoms or abnormal radiology warrant further investigation for other pathologies, particularly HIV infection, and repeat culture of sputum and other relevant samples.

*M. avium* complex was particularly common among symptomatic individuals in the clinic patient group, who had high prevalence of HIV infection.* M. avium* complex is known to be common where it is sought and appropriate diagnostics used in HIV-positive populations and disease is associated with low CD4 cell counts [29–32]. These individuals in particular require further investigation and specific treatment, with early antiretroviral therapy and cotrimoxazole prophylaxis. It is noteworthy that 30/38* M. avium* complex isolates grew in liquid but not solid culture media and none of these 30 were smear positive, suggesting that* M. avium* complex infection may be underdiagnosed in settings of high HIV prevalence where liquid culture medium is not used. Pulmonary isolates may reflect disseminated disease and liquid culture will be needed to diagnose this infection in settings where the Xpert MTB/RIF assay is used as first line diagnostic test for tuberculosis. There was a notable absence of* M. avium*, with other* M. avium* complex subspecies found (*M. colombiense*,* M. vulneris*, and* M. intracellulare*). This may reflect immunosuppression, as* M. colombiense* is closely related to* M. avium* and has been isolated from HIV-positive individuals previously [33] or may reflect geographical variation in NTM species [34].

Mining populations have long been known to be at risk of NTM infection and disease [12] and to some extent results presented here are relevant primarily to this population. However, comparisons of species distribution, HIV prevalence, and clinical features are relevant to other settings in which HIV and tuberculosis are prevalent and liquid mycobacterial culture media are used. Similar results have been seen in Southeast Asia in a study showing high prevalence of NTM isolated by liquid culture of sputum among HIV-positive individuals, with* M. kansasii* most frequent in those with pulmonary disease and* M. avium* complex among those with disseminated disease [35].

The clinic patient group was restricted to individuals with no prior tuberculosis treatment, in order to maximise the impact of the parent laboratory study on routine health services. In addition, the clinic patient group was more symptomatic than the pre-IPT screening group and there are likely to have been differences in disease spectrum between these groups. For this reason, characteristics of the two groups are presented separately. In addition, despite excluding individuals with* M. tuberculosis* isolated from other specimens within six months of the NTM, we cannot definitively exclude culture negative tuberculosis.

ATS criteria for lung disease due to NTM [19] include, amongst other criteria shown in Table 6, a requirement for NTM to be isolated from expectorated sputum samples on more than one occasion. We were not able to apply these criteria in this study because of its retrospective design. This is a limitation of this study. However, authors of the ATS criteria, primarily intended to guide diagnosis of lung disease, are clear that they were written with the United States setting in mind and are not validated in other settings. Among miners in South Africa, these criteria have been shown to be difficult to apply even in prospective studies, because of the requirement for repeated sampling and detailed imaging and frequent use of presumptive antimycobacterial treatment [10] in a programme designed for tuberculosis detection and treatment. Such repeat intensive laboratory diagnostics are not part of the national guidelines for tuberculosis control in South Africa [36].

## 5. Conclusions

In conclusion,* M. avium* complex, largely found among symptomatic, smear-negative individuals, will be underdiagnosed where liquid culture is not used. Newer culture techniques are advisable wherever feasible, although there are of course cost and infrastructure obstacles to widespread use. Key pathogenic organisms should be identified where possible, so that specific treatment can be given if required. HIV testing coverage was poor and prevalence among those with known HIV status was high. Improved HIV testing strategies are required for those being investigated for tuberculosis in this and other settings of high HIV prevalence.

## Figures and Tables

**Table 1:** Species identified using 16S sequencing, with smear status, HIV status, and CD4 counts (*n* = 232).

Species	Number isolated (% of group)	Number (%) smear positive	Number with mixed species^4^	HIV prevalence^1^ (%)	Median CD4^2^ (cells/*µ*L) (range (number included))
Clinic patient group					
* M. avium *complex	25 (26)	2 (8)	5	8/10	87 (14, 827 [5])
* M. kansasii *	21 (22)	6 (29)	3	5/8	85 (39, 763 [5])
* M. parascrofulaceum *	9 (9)	1 (11)	0	3/3	102 (66, 138 [2])
* M. fortuitum *	9 (9)	2 (22)	1	1/1	[0]
* M. gordonae *	7 (7)	0	1	1/1	195 [1]
New species	7 (7)	1 (14)	0	3/3	475 (236, 512 [3])
Other species	18 (19)	0	2	4/5	291.5 (154, 492 [4])

Total	96	12 (12.5)	12 (12.5% of 96 isolates)	25/31 (80.6)	215.5 (14, 827 [20])

Pre-IPT^3^ screening group					
* M. gordonae *	53 (39)	8 (15)	5	10/12	298 (66, 396 [8])
* M. kansasii *	29 (21)	11 (38)	1	9/14	183.5 (69, 544 [8])
* M. avium *complex	13 (10)	0	3	4/5	211 (92, 534 [4])
* M. parascrofulaceum *	12 (9)	2 (17)	1	1/2	223 [1]
* M. fortuitum *	11 (8)	0	1	3/5	358 (245, 471 [2])
* M. szulgai *	5 (4)	0	2	2/2	132.5 (123, 142 [2])
New species	3 (2)	0	0	0/0	[0]
Other species	10 (7)	0	2	3/3	311 (252, 370 [2])

Total	136	21 (15.4)	15 (11.0% of 136 isolates)	32/43 (74.4)	245 (66, 544 [27])

^1^Fisher's exact test for difference in HIV prevalence by species, both groups combined, *P* = 0.79.

^2^Median CD4 count by species for those known to be HIV-positive, combining both groups (clinic patients and IPT screening group) and including 8 species compared using Kruskal-Wallis test, which showed no evidence for difference by species, *P* = 0.42.

^3^IPT: isoniazid preventive therapy.

^4^For specimens containing mixed NTM species on PCR sequencing, the main species is reported; minority species were not identified, but the numbers with mixed species are given here. This does not apply to those with concurrent *M. tuberculosis*, who were excluded from this study.

**Table 2:** Species distribution by HIV status, among 74 individuals with known status.

Species	HIV-positive (total = 57) *n* (%)	HIV-negative (total = 17) *n* (%)
*M. kansasii *	14 (25)	8 (47)
*M. avium *complex	12 (21)	3 (18)
* M. colombiense *	5	1
* M. intracellulare *	4	1
* M. vulneris *	3	1
*M. gordonae *	11 (19)	2 (12)
*M. fortuitum *	4 (7)	2 (12)
*M. parascrofulaceum *	4 (7)	1 (6)
*M. szulgai *	2 (4)	0
New species	3 (5)	0
Unknown	7 (12)	1 (6)

**Table 3:** Symptoms reported by route of recruitment to study and species of nontuberculous mycobacterium isolated (*n* = 232).

Species	Number isolated	Number reporting cough *n* (%)	Number reporting night sweats^1^ *n* (%)	Number reporting haemoptysis *n* (%)	Number reporting weight loss^2^ *n* (%)	Number reporting fever *n* (%)	Number reporting any symptom *n* (%)	Number with chest radiograph classified as definite or possible active tuberculosis *n*/number with chest radiograph available
Clinic patient group								
* M. avium *complex	25	17 (68)	13 (52)	4 (16)	11 (44)	11 (44)	19 (76)	2/14
* M. kansasii *	21	11 (52)	9 (43)	1 (5)	10 (48)	9 (43)	14 (67)	4/9
* M. parascrofulaceum *	9	8 (89)	7 (78)	0	8 (89)	6 (67)	9 (100)	1/7
* M. fortuitum *	9	7 (78)	5 (56)	0	5 (56)	3 (33)	7 (78)	1/5
* M. gordonae *	7	5 (71)	2 (29)	0	4 (57)	4 (57)	6 (86)	1/3
New species	7	5 (71)	3 (43)	1 (14)	2 (29)	4 (57)	5 (71)	0/5
Other species	18	13 (72)	11 (61)	3 (17)	7 (39)	10 (56)	15 (83)	1/7

Total	96	66 (69)	50 (52.1)	9 (9.4)	47 (49.0)	47 (49.0)	75 (78.1)	10/50

Pre-IPT^3^ screening group								
* M. gordonae *	53	26 (49)	12 (23)	§	17 (32)	§	34 (64)	13/46
* M. kansasii *	29	8 (28)	2 (7.0)	§	6 (21)	§	12 (41)	13/25
* M. avium *complex	13	8 (62)	5 (39)	§	3 (23)	§	9 (69)	1/12
* M. parascrofulaceum *	12	2 (17)	2 (17)	§	4 (33)	§	6 (50)	4/11
* M. fortuitum *	11	5 (46)	3 (27)	§	1 (9)	§	5 (46)	5/10
* M. szulgai *	5	4 (80)	1 (20)	§	2 (40)	§	4 (80)	2/5
New species	3	1 (33)	1 (33)	§	1 (33)	§	1 (33)	1/2
Other species	10	5 (50)	1 (10)	§	5 (50)	§	6 (60)	1/10

Total	136	59 (43.4)	27 (19.9)	§	39 (28.7)	§	77 (56.6)	40/121

^1^Night sweats were defined by the wording of the question put to study participants: “Do you have drenching night sweats? (Sweat so much at night that clothes/pillows are soaking wet?).”

^2^Weight loss was defined by the wording of the question put to study participants: “Do you have unintentional weight loss? (In the last 6 months have your clothes become looser?).”

^3^IPT: isoniazid preventive therapy.

^§^Data not available as questionnaire used in pre-IPT screening for tuberculosis did not include this question.

**Table 4:** Deaths and medical separations, from recruitment until end of 2010, median 32 months (*n* = 218).

NTM species	Death *n* (row %)	Medical separation *n* (row %)	Total number of individuals
*M. gordonae *	0	13 (26)	51
*M. kansasii *	2 (4)	16 (33)	49
*M. avium *complex	1 (3)	12 (32)	38
*M. parascrofulaceum *	2 (11)	2 (11)	19
*M. fortuitum *	1 (5)	11 (58)	19
*M. szulgai *	0	1 (20)	5
New species	1 (10)	1 (10)	10
Other species	3 (11)	7 (26)	27

Total	10 (4.6)	63 (28.9)	218

**Table 5:** Probability of remaining in the workforce during follow-up for individuals with the three predominant NTM species.

Species	6 months (%, 95% CI)	12 months (%, 95% CI)	24 months (%, 95% CI)
*M. gordonae *	93.8 (82.0–98.0)	85.0 (91.0–92.5)	80.3 (65.5–89.3)
*M. kansasii *	87.3 (73.9–94.1)	78.4 (63.5–87.8)	69.2 (53.6–80.5)
*M. avium *complex	88.3 (71.7–95.4)	81.7 (63.7–91.4)	72.9 (52.9–94.4)

**Table 6:** American Thoracic Society criteria for lung disease due to NTM (adapted from [19]).

Criteria for diagnosis of lung disease due to NTM	
Clinical	(1) Pulmonary symptoms, nodular or cavitary opacities on chest radiograph, or an HRCT scan that shows multifocal bronchiectasis with multiple small nodules And (2) Appropriate exclusion of other diagnoses

Microbiological	(1) Positive culture results from at least two separate expectorated sputum samples Or (2) Positive culture results from at least one bronchial wash or lavage Or (3) Transbronchial o other lung biopsy with mycobacterial histopathological features (granulomatous inflammation or AFB) and positive culture for NTM or biopsy showing mycobacterial histopathological features and one or more sputum or bronchial washings that are culture positive for NTM
